# Supplementary figures and images for: Evolutionary gain and loss of a plant pattern-recognition receptor for HAMP recognition
Source: eLife. 2022 Nov 15;11:e81050. doi: 10.7554/eLife.81050 (PMC9718524; doi:10.7554/eLife.81050)

Figure 3-figure supplement 1-source data

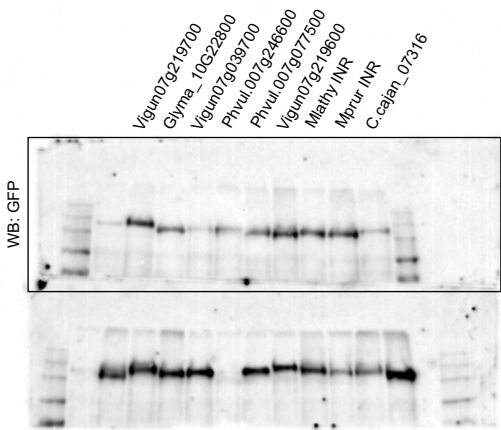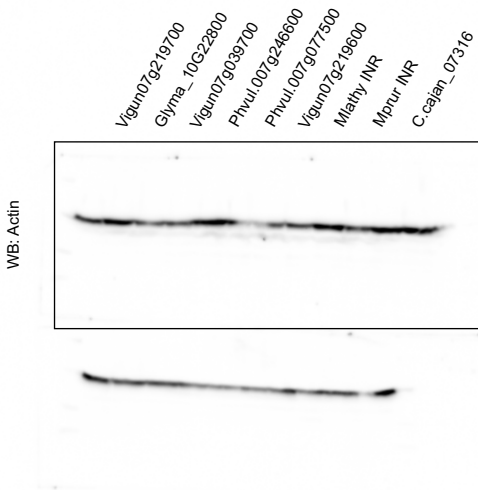

Supplement: Figure 3—figure supplement 1—source data 1. — For the western blot of C-terminal GFP tagged INR and INR-like homolog receptors, after wet transfer, nitrocellulose membranes were physically split at 75 kDA and probed separately with (1) GFP antibody (>75 kDa) and (2) actin antibody as a loading control (<75 kDa), resulting in two tiff files for each panel. [file elife-81050-fig3-figsupp1-data1.zip › Figure 3-figure supplement 1-source data 1/Figure 3-figure supplement 1-source data 1.pdf]

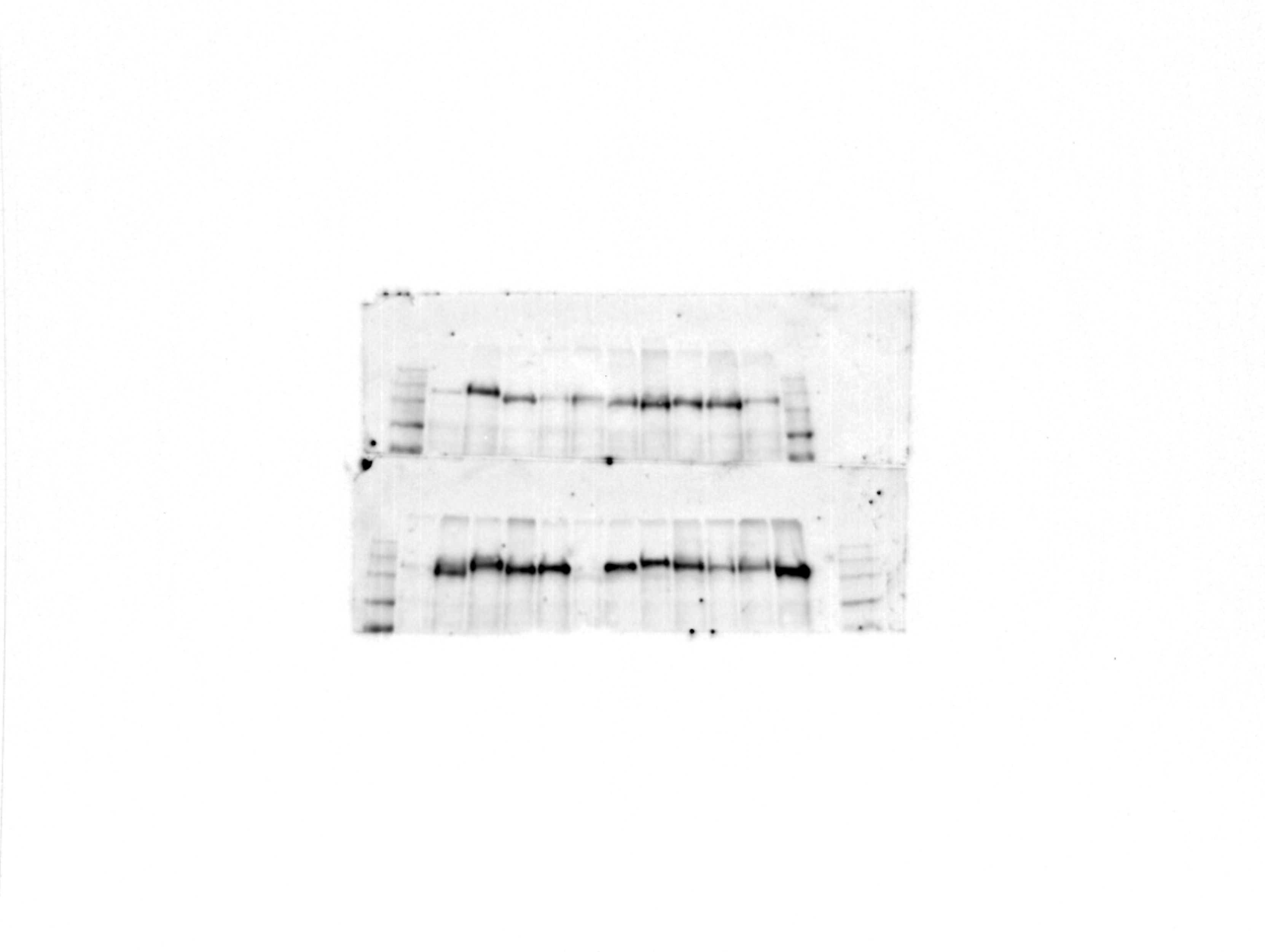

Supplement: Figure 3—figure supplement 1—source data 1. — For the western blot of C-terminal GFP tagged INR and INR-like homolog receptors, after wet transfer, nitrocellulose membranes were physically split at 75 kDA and probed separately with (1) GFP antibody (>75 kDa) and (2) actin antibody as a loading control (<75 kDa), resulting in two tiff files for each panel. [file elife-81050-fig3-figsupp1-data1.zip › Figure 3-figure supplement 1-source data 1/S1F10-0128-184955_SF3a_GFP.tif]

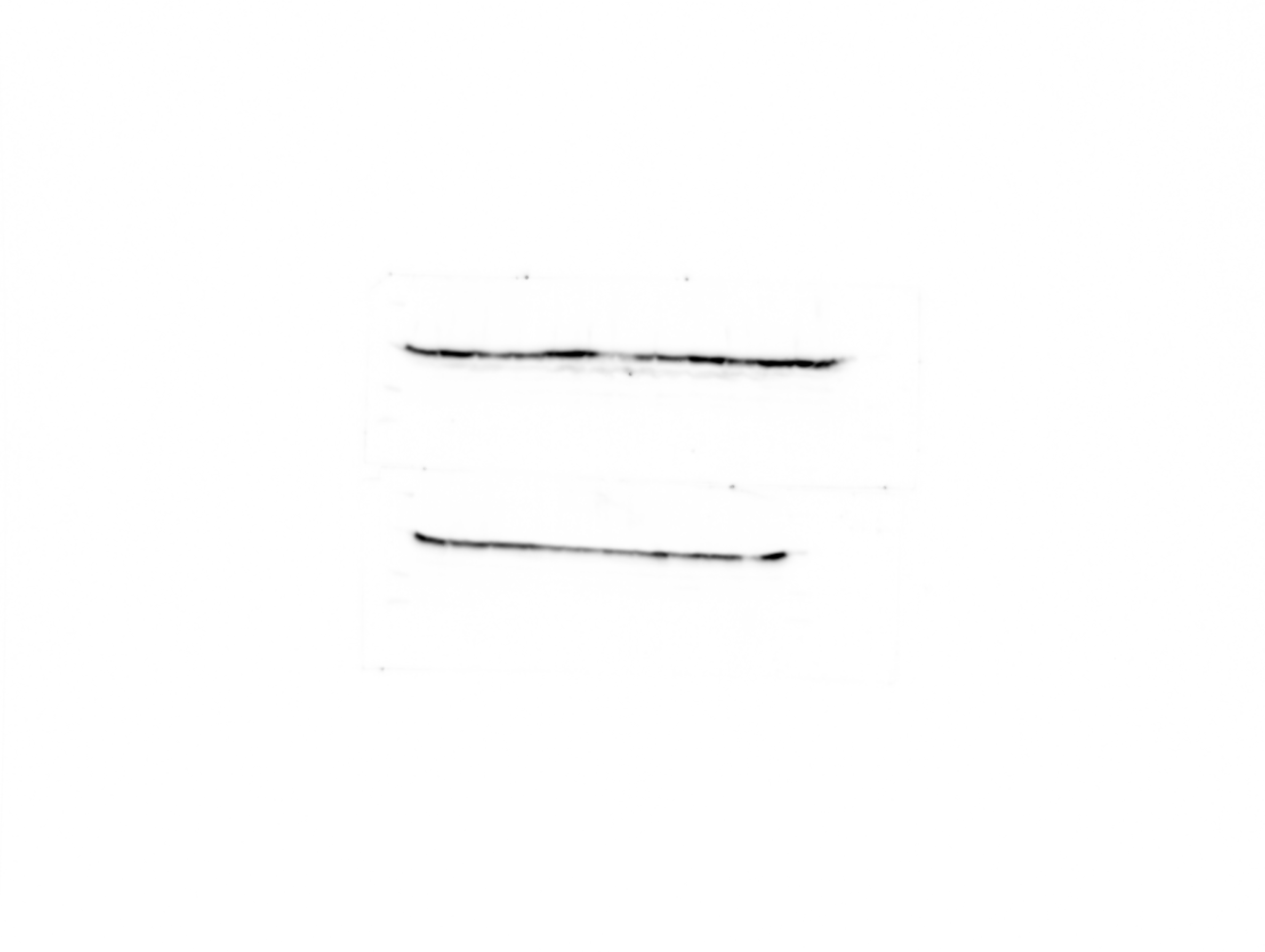

Supplement: Figure 3—figure supplement 1—source data 1. — For the western blot of C-terminal GFP tagged INR and INR-like homolog receptors, after wet transfer, nitrocellulose membranes were physically split at 75 kDA and probed separately with (1) GFP antibody (>75 kDa) and (2) actin antibody as a loading control (<75 kDa), resulting in two tiff files for each panel. [file elife-81050-fig3-figsupp1-data1.zip › Figure 3-figure supplement 1-source data 1/S2F5-0128-185212_SF3a_Actin.tif]

Figure 4-figure supplement 1-source data 1

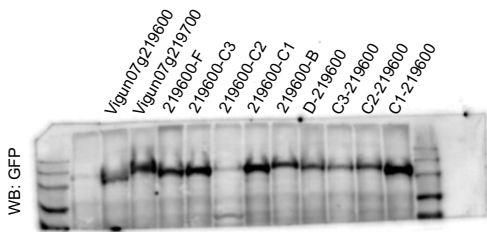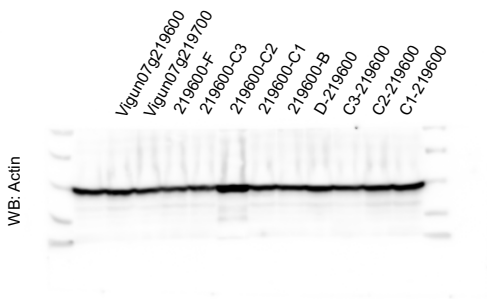

Supplement: Figure 4—figure supplement 1—source data 1. — For the western blot of C-terminal GFP tagged chimeric receptors, after wet transfer, nitrocellulose membranes were physically split at 75 kDA and probed separately with (1) GFP antibody (>75 kDa) and (2) actin antibody as a loading control (<75 kDa), resulting in two tiff files for each panel. [file elife-81050-fig4-figsupp1-data1.zip › Figure 4-figure supplement 1-source data 1/Figure 4-figure supplement 1-source data 1.pdf]

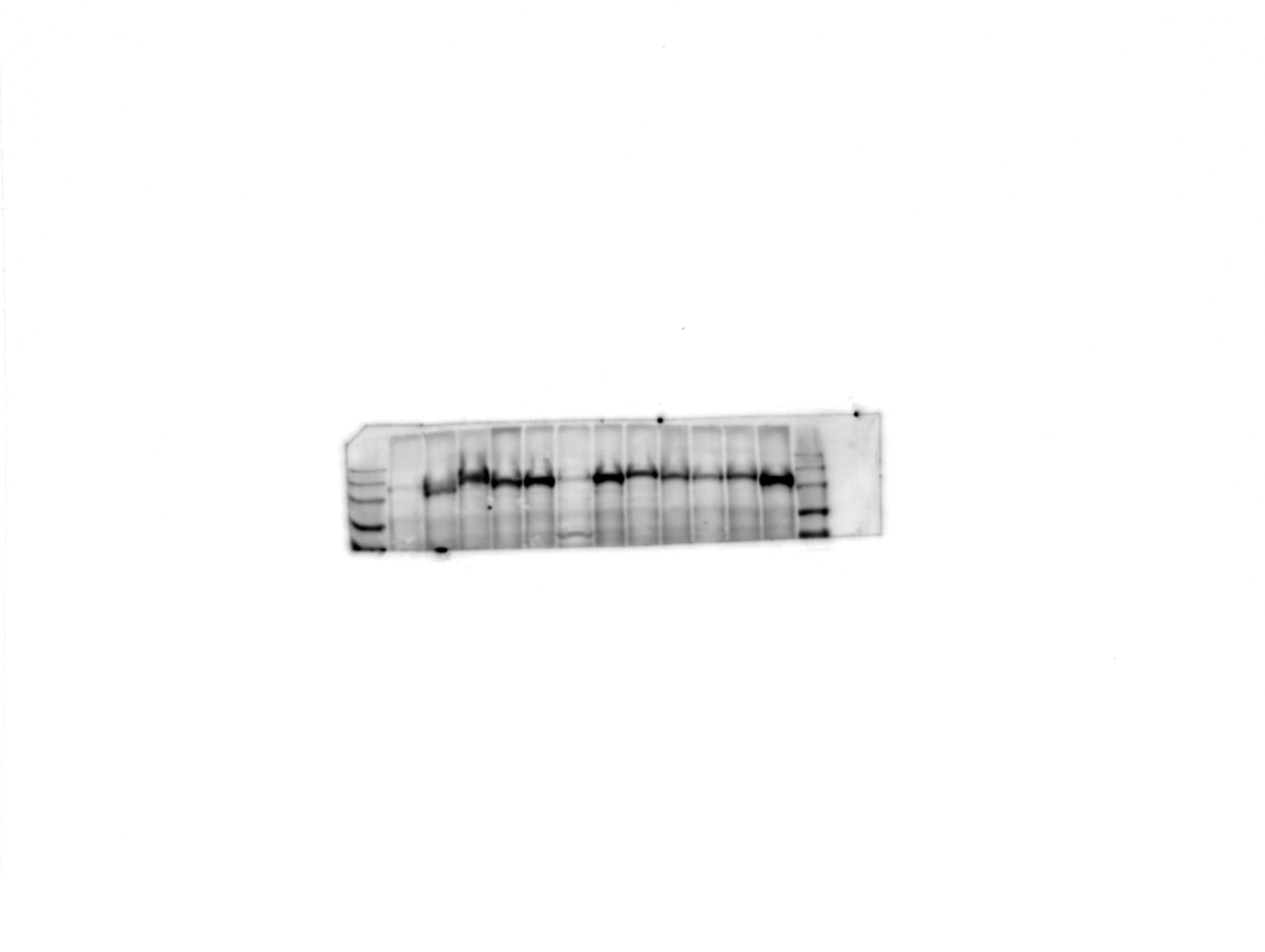

Supplement: Figure 4—figure supplement 1—source data 1. — For the western blot of C-terminal GFP tagged chimeric receptors, after wet transfer, nitrocellulose membranes were physically split at 75 kDA and probed separately with (1) GFP antibody (>75 kDa) and (2) actin antibody as a loading control (<75 kDa), resulting in two tiff files for each panel. [file elife-81050-fig4-figsupp1-data1.zip › Figure 4-figure supplement 1-source data 1/S1F2-0130-131630_SF3b_GFP.tif]

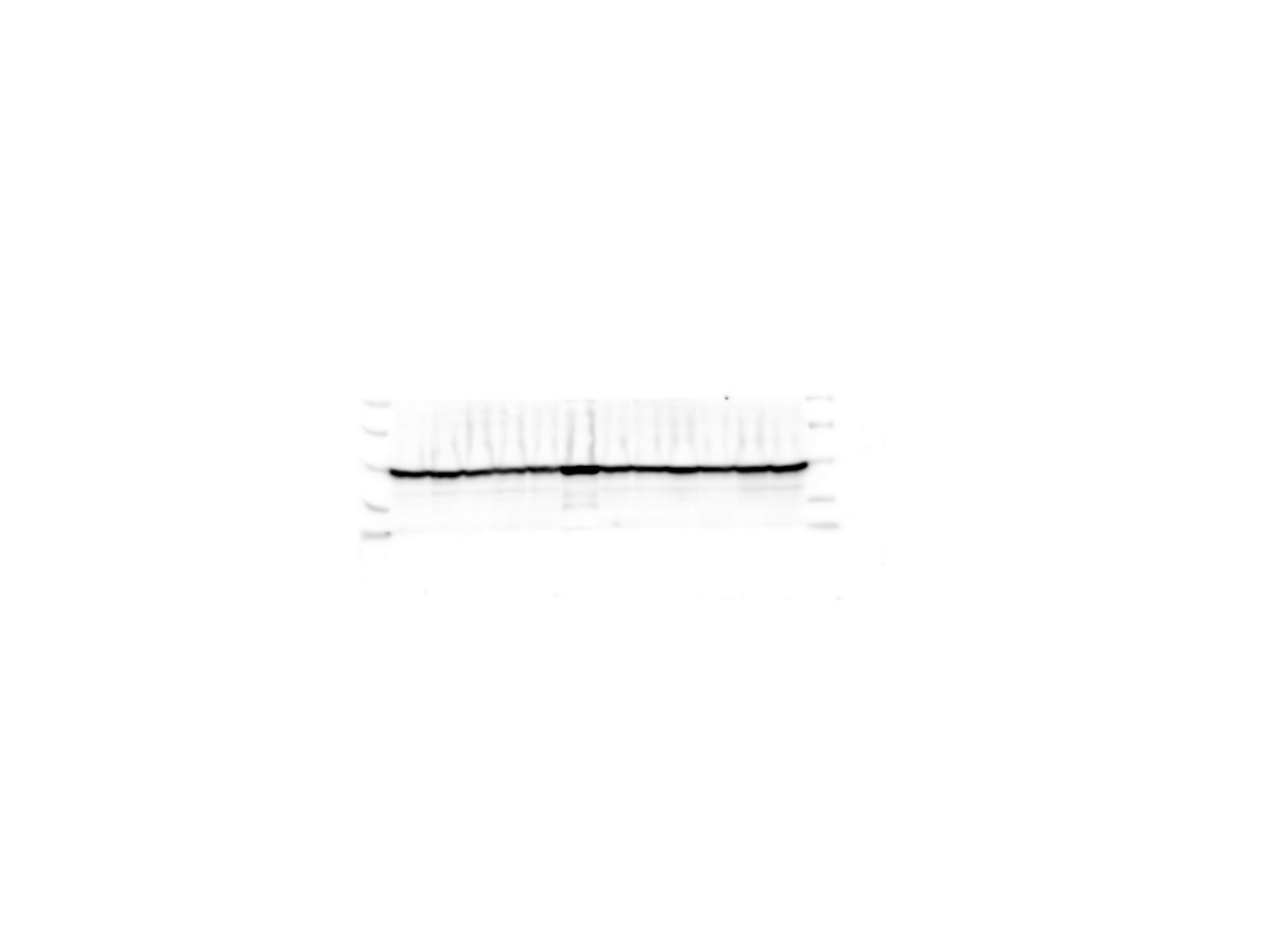

Supplement: Figure 4—figure supplement 1—source data 1. — For the western blot of C-terminal GFP tagged chimeric receptors, after wet transfer, nitrocellulose membranes were physically split at 75 kDA and probed separately with (1) GFP antibody (>75 kDa) and (2) actin antibody as a loading control (<75 kDa), resulting in two tiff files for each panel. [file elife-81050-fig4-figsupp1-data1.zip › Figure 4-figure supplement 1-source data 1/S2F4-0130-132618_SF3b_Actin.tif]

Figure 5-figure supplement 2-source data 1

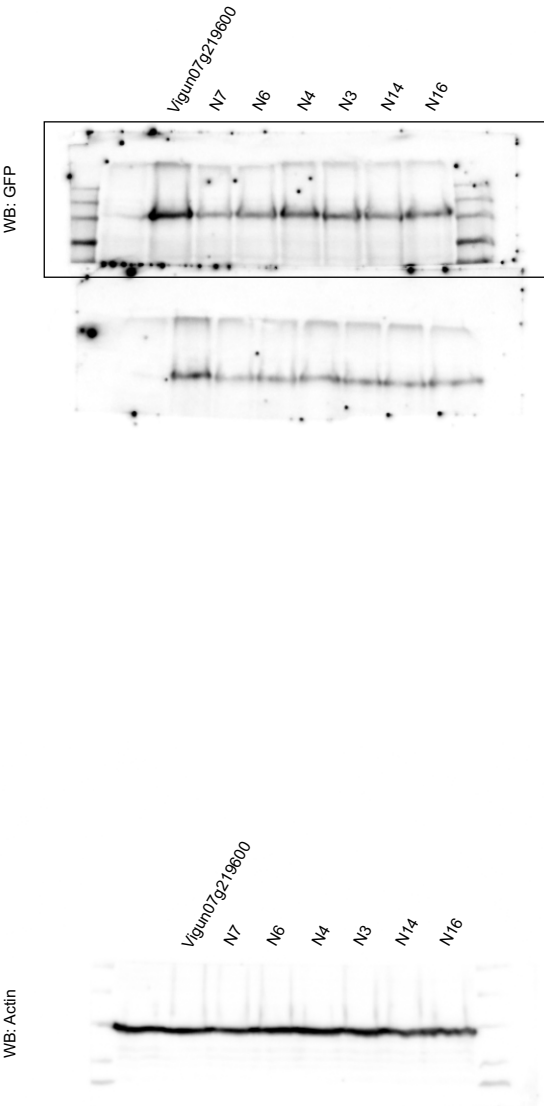

Supplement: Figure 5—figure supplement 2—source data 1. — For the western blot of C-terminal GFP tagged ASR receptors, after wet transfer, nitrocellulose membranes were physically split at 75 kDA and probed separately with (1) GFP antibody (>75 kDa) and (2) actin antibody as a loading control (<75 kDa), resulting in two tiff files for each panel. [file elife-81050-fig5-figsupp2-data1.zip › Figure 5-figure supplement 2-source data 1/Figure 5-figure supplement 2-source data 1.pdf]

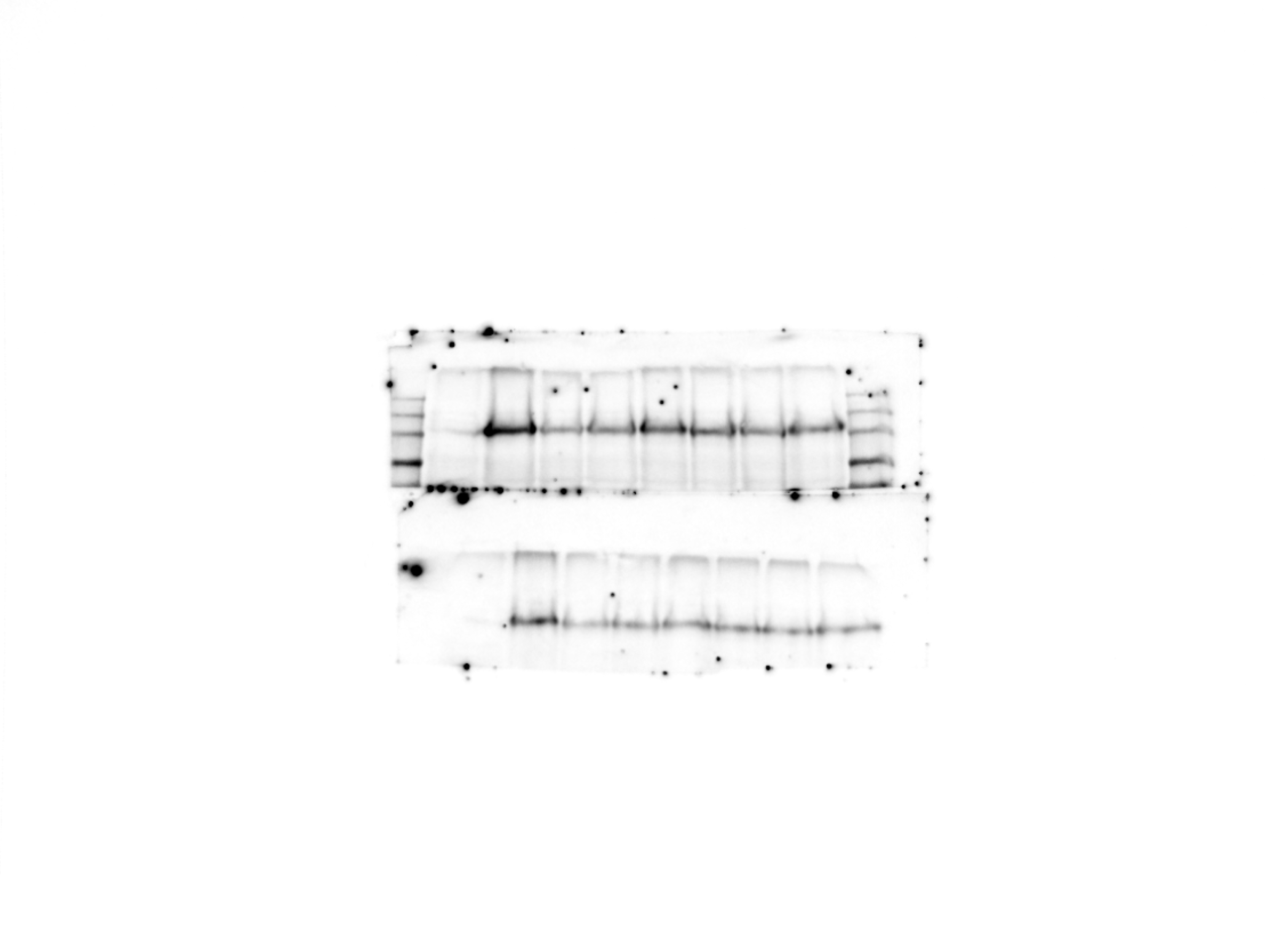

Supplement: Figure 5—figure supplement 2—source data 1. — For the western blot of C-terminal GFP tagged ASR receptors, after wet transfer, nitrocellulose membranes were physically split at 75 kDA and probed separately with (1) GFP antibody (>75 kDa) and (2) actin antibody as a loading control (<75 kDa), resulting in two tiff files for each panel. [file elife-81050-fig5-figsupp2-data1.zip › Figure 5-figure supplement 2-source data 1/S1F1-0215-125551_SF3c_GFP.tif]

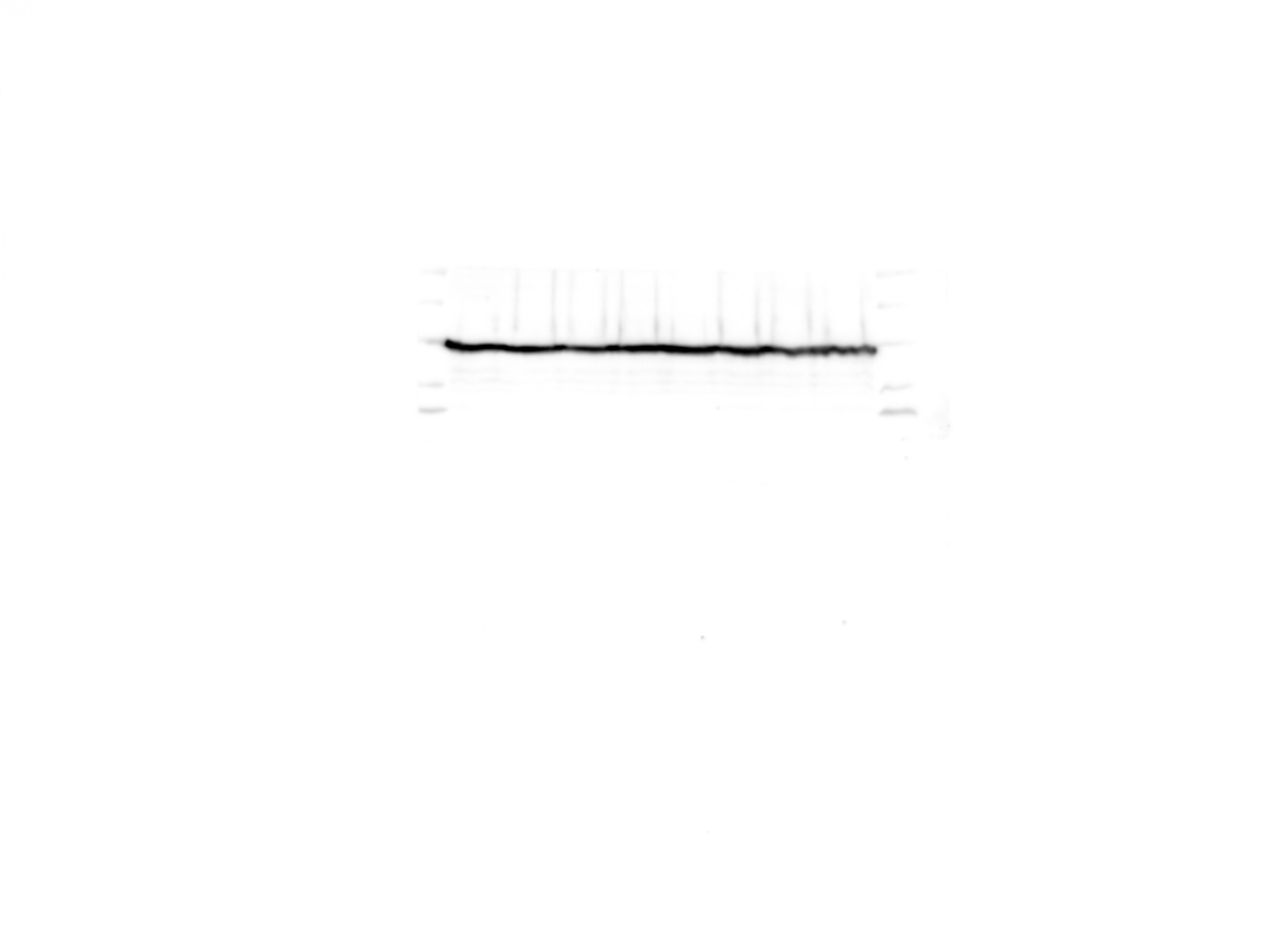

Supplement: Figure 5—figure supplement 2—source data 1. — For the western blot of C-terminal GFP tagged ASR receptors, after wet transfer, nitrocellulose membranes were physically split at 75 kDA and probed separately with (1) GFP antibody (>75 kDa) and (2) actin antibody as a loading control (<75 kDa), resulting in two tiff files for each panel. [file elife-81050-fig5-figsupp2-data1.zip › Figure 5-figure supplement 2-source data 1/S2F1-0215-130130_SF3c_Actin.tif]

Figure 6-figure supplement 1-source data 1

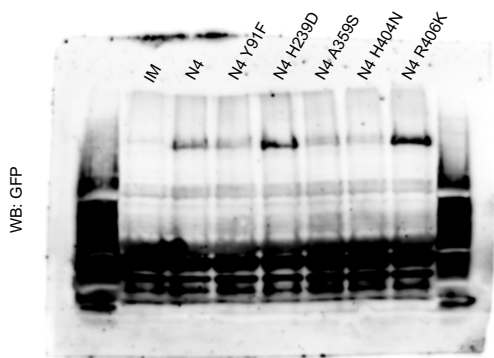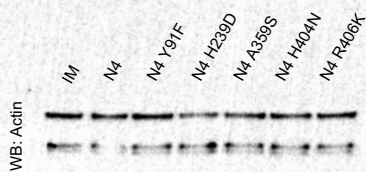

Supplement: Figure 6—figure supplement 1—source data 1. — Two identical western blots were run in parallel of the C-terminal GFP tagged N4 and N4 variant constructs receptors and probed differentially with (1) GFP antibody and (2) actin antibody (A0480) as a loading control, resulting in two tiff files for each blot. [file elife-81050-fig6-figsupp1-data1.zip › Figure 6-figure supplement 1-source data 1/Figure 6-figure supplement 1-source data 1.pdf]

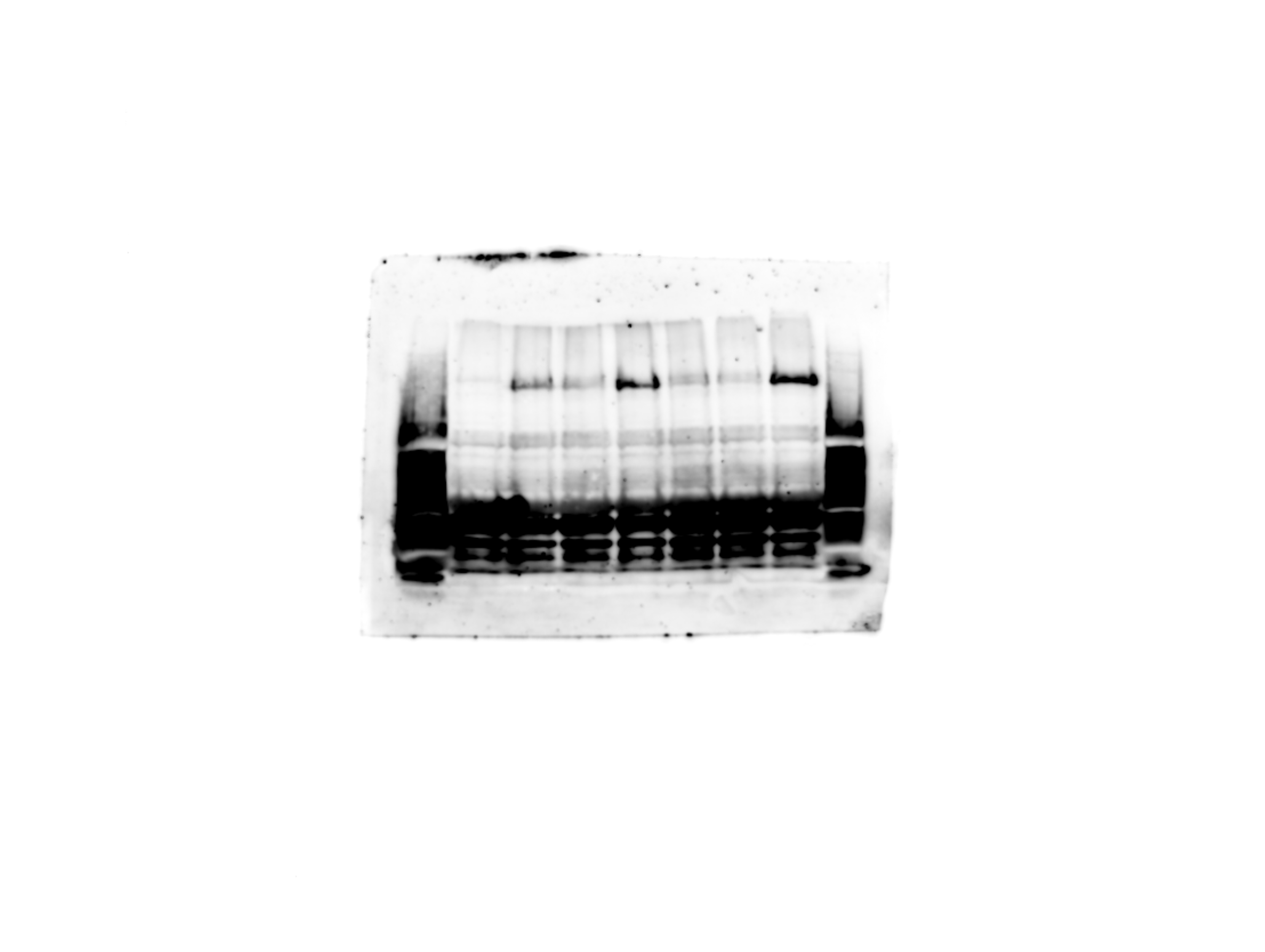

Supplement: Figure 6—figure supplement 1—source data 1. — Two identical western blots were run in parallel of the C-terminal GFP tagged N4 and N4 variant constructs receptors and probed differentially with (1) GFP antibody and (2) actin antibody (A0480) as a loading control, resulting in two tiff files for each blot. [file elife-81050-fig6-figsupp1-data1.zip › Figure 6-figure supplement 1-source data 1/S1F9-0816-085652_pub.tif]

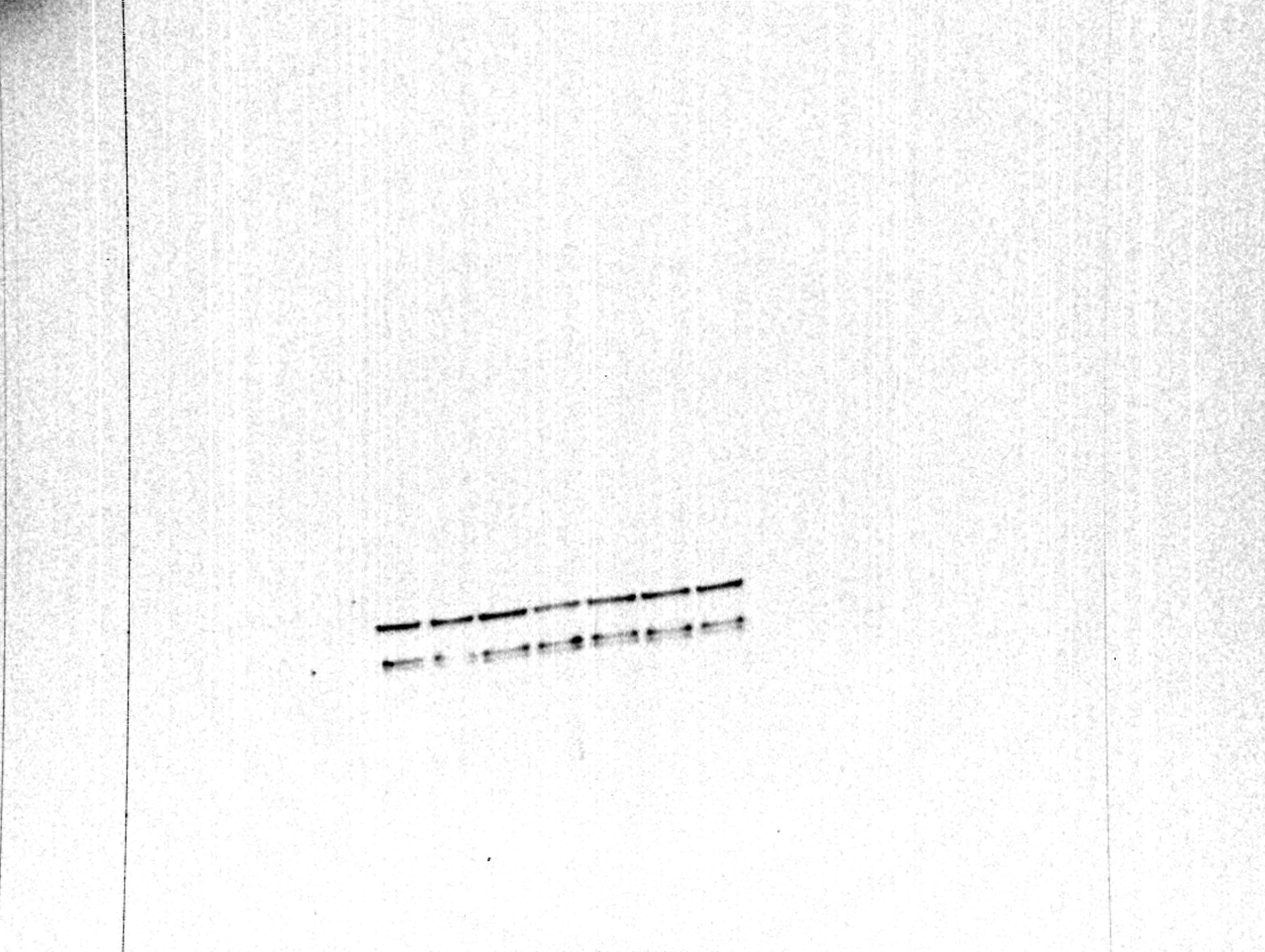

Supplement: Figure 6—figure supplement 1—source data 1. — Two identical western blots were run in parallel of the C-terminal GFP tagged N4 and N4 variant constructs receptors and probed differentially with (1) GFP antibody and (2) actin antibody (A0480) as a loading control, resulting in two tiff files for each blot. [file elife-81050-fig6-figsupp1-data1.zip › Figure 6-figure supplement 1-source data 1/S2F8-0816-102009_pub.tif]
