# Supplementary material for: Evolutionary gain and loss of a plant pattern-recognition receptor for HAMP recognition
Source: eLife. 2022 Nov 15;11:e81050. doi: 10.7554/eLife.81050 (PMC9718524; doi:10.7554/eLife.81050)
Supplement: Supplementary file 8. — Differences or consensus (dashed marks) from ancestral sequence reconstruction (ASR) construct N3 are indicated for all other sequences. The five AA polymorphisms between N3 and N4 that are similar within all LRR-RLPs of the INR clade and different in LRR-RLPs of the INR-like 1 sister clade are highlighted in yellow. C1-domain starts at the N-terminus, C3-domain ends at the C-terminus, C1 and C3 domain are separated by the C2-domain which is highlighted in the frame. [file elife-81050-supp8.pdf]

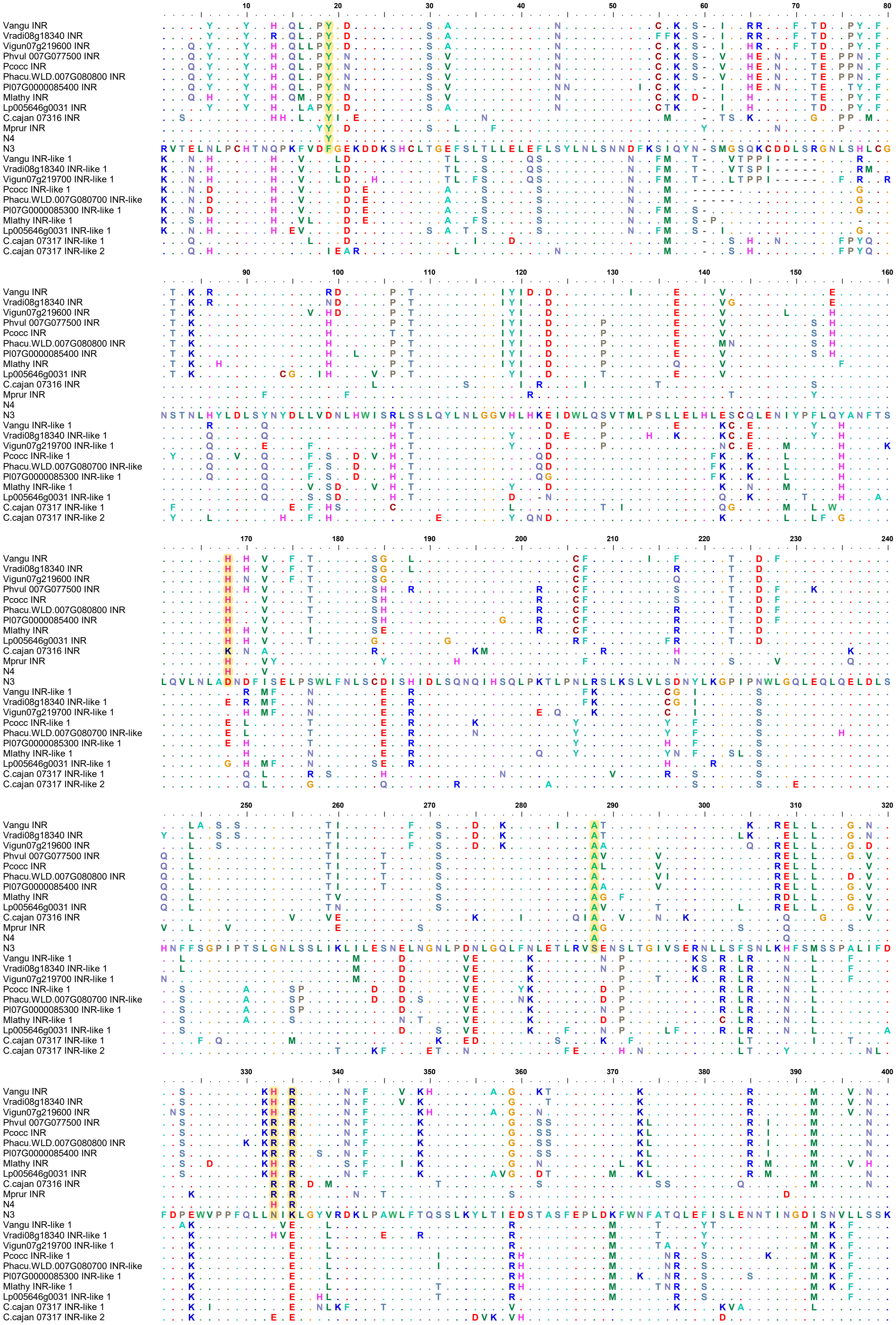
Vangu INR
Vradi08g18340 INR
Vigun07g219600 INR
Phvul 007G077500 INR
Pcocc INR
Phacu.WLD.007G080800 INR
Pl07G0000085400 INR
Mlathy INR
Lp005646g0031 INR
C.cajan 07316 INR
Mprur INR
N4
N3
Vangu INR-like 1
Vradi08g18340 INR-like 1
Vigun07g219700 INR-like 1
Pcocc INR-like 1
Phacu.WLD.007G080700 INR-like
Pl07G0000085300 INR-like 1
Mlathy INR-like 1
Lp005646g0031 INR-like 1
C.cajan 07317 INR-like 1
C.cajan 07317 INR-like 2

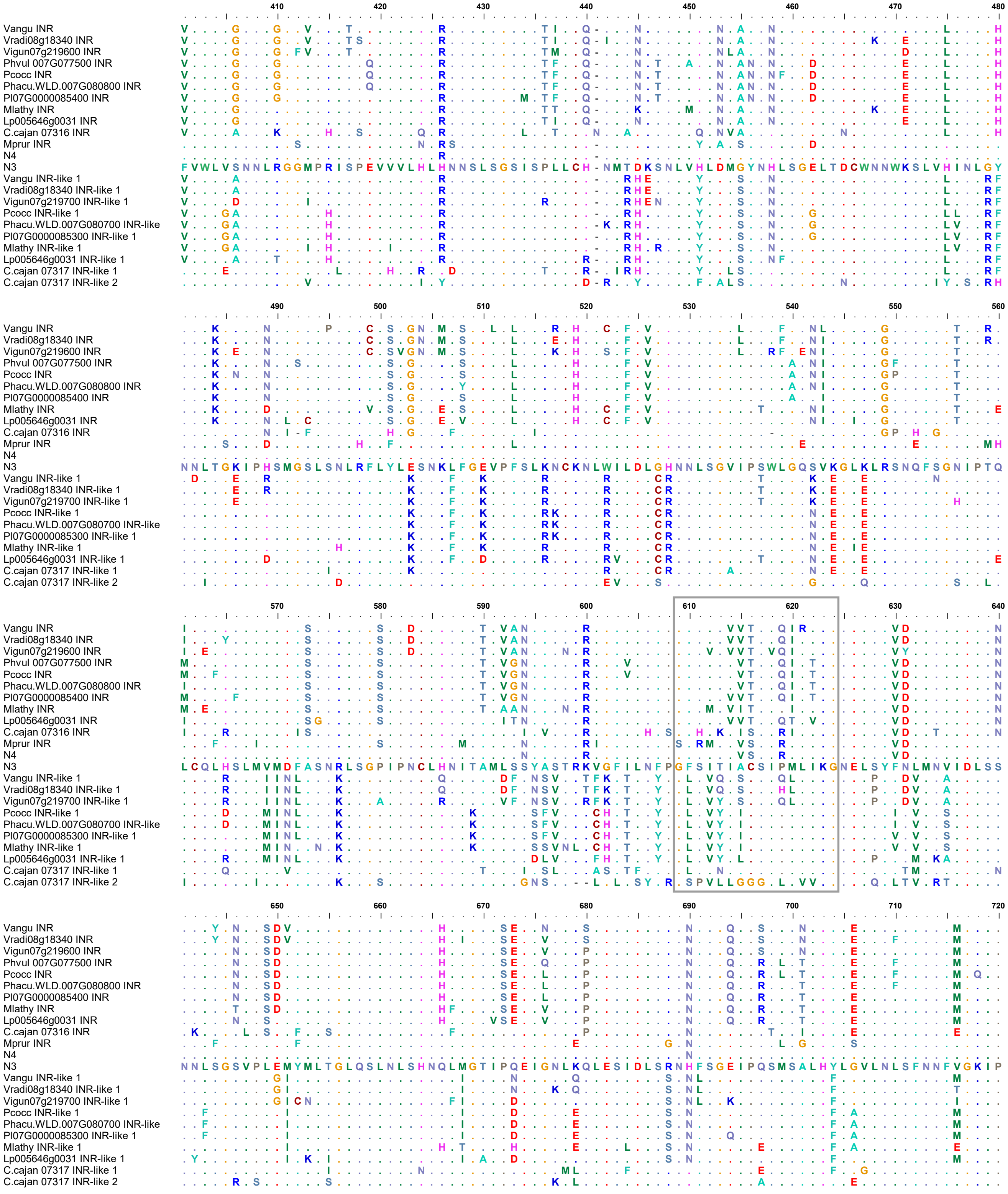
410
420
430
440
450
460
470
480
490
500
510
520
530
540
550
560
570
580
590
600
610
620
630
640
650
660
670
680
690
700
710
720
Vangu INR
Vradi08g18340 INR
Vigun07g219600 INR
Phvul 007G077500 INR
Pcocc INR
Phacu.WLD.007G080800 INR
Pl07G0000085400 INR
Mlathy INR
Lp005646g0031 INR
C.cajan 07316 INR
Mprur INR
N4
N3
Vangu INR-like 1
Vradi08g18340 INR-like 1
Vigun07g219700 INR-like 1
Pcocc INR-like 1
Phacu.WLD.007G080700 INR-like
Pl07G0000085300 INR-like 1
Mlathy INR-like 1
Lp005646g0031 INR-like 1
C.cajan 07317 INR-like 1
C.cajan 07317 INR-like 2
